# The FGF-1-specific single-chain antibody scFv1C9 effectively inhibits breast cancer tumour growth and metastasis

**DOI:** 10.1111/jcmm.12371

**Published:** 2014-08-15

**Authors:** Hengliang Shi, Chunling Fu, Wei Wang, Yu Li, Shuang Du, Rangjuan Cao, Jingying Chen, Dong Sun, Zhongyu Zhang, Xingzhi Wang, Xiaojuan Zhu

**Affiliations:** 1 Key Laboratory of Molecular Epigenetics of the Ministry of Education, Institute of Cytology and Genetics, Northeast Normal UniversityChangchun, China; 2 Department of Neurosurgery, Affiliated Hospital of Xuzhou Medical CollegeXuzhou, China; 3 Department of Hematology, Affiliated Hospital of Xuzhou Medical CollegeXuzhou, China

**Keywords:** FGF-1, lung metastasis, scFv1C9, tumour growth, tumourigenesis

## Abstract

Immunotherapy mediated by recombinant antibodies is an effective therapeutic strategy for a variety of cancers. In a previous study, we demonstrated that the fibroblast growth factor 1 (FGF-1)-specific recombinant antibody scFv1C9 arrests the cell cycle at the G0/G1 transition by blocking the intracrine FGF-1 pathway in breast cancer cells. Here, we further show that the overexpression of scFv1C9 in MCF-7 and MDA-MB-231 breast cancer cells by lentiviral infection resulted in decreased tumourigenicity, tumour growth and lung metastasis through FGF-1 neutralization. We found that scFv1C9 resulted in the up-regulation of p21, which in turn inhibited the expression of CDK2 and blocked cell cycle progression. To explore the potential role of scFv1C9 *in vivo*, we delivered the gene into solid tumours by electroporation, which resulted in significant inhibition of tumour growth. In tumour tissue sections, immunohistochemical staining of the cellular proliferation marker Ki-67 and the microvessel marker CD31 showed a reduction in the proliferative index and microvessel density, respectively, upon expression of scFv1C9 compared with the appropriate controls. Thus, our data indicate a central role for scFv1C9 in blocking the intracrine pathway of FGF-1, therefore, scFv1C9 could be developed in an effective therapeutic for breast cancer.

## Introduction

Fibroblast growth factors (FGFs) constitute a superfamily of polypeptides that are involved in multiple biological processes during embryo development, wound healing and angiogenesis 1. Most FGFs are synthesized with either a cleavable or non-cleavable N-terminal hydrophobic signal peptide and are secreted from the cell *via* the endoplasmic reticulum-Golgi pathway 2,3. The activities of FGFs are usually mediated by FGF receptors (FGFRs) which have tyrosine kinase activities 4. FGF-1 is expressed in a wide variety of cell types and functions as a proliferation, differentiation and survival factor 5. As it does not possess a signal peptide and does contain a nuclear localization sequence (NLS), FGF-1 is consistently located cytoplasm and in the cell nucleus under normal conditions 6,7. Various types of stress, such as heat shock, hypoxia and serum starvation, induce the release of FGF-1 from cells 8–10. FGF-1 stimulates the development of several types of cancers, including bladder cancer, hepatocellular carcinoma, pancreatic cancer and breast cancer 11–13, which suggests that FGF-1 signalling is a potential target for cancer therapy. Thus, blocking FGF signalling might be an effective method for cancer therapy. PD173074 (1-*tert*-butyl-3-[6-(3,5-dimethoxy-phenyl)-2-(4-diethylaminobutylamino)-pyridol[2,3-d]pyrimidin-7-yl]-urea) has been shown to selectively inhibit FGFR autophosphorylation and therefore tyrosine kinase activity, resulting in the growth arrest of breast cancer cells in G1 14,15.

Antibody-mediated immunotherapy is an effective way to extend the life of cancer patients who are reliant on a particular variant of FGFR 16. Monoclonal antibodies specific for FGFR1, which block FGF signalling, inhibit the growth of tumour cells; however, problems remain, such as limited antibody uptake at the tumour site and the high immunogenicity of full-length antibodies, both of which decrease the anti-tumour effects of these antibodies 17. Single-chain variable fragment (scFv) monoclonal antibodies, which have a lower molecular weight than full-length antibodies, are increasingly being used as therapeutic effectors in clinical applications. A recombinant scFv specific for FGFR3 has been demonstrated to inhibit the proliferation of bladder cancer and myeloma 18,19. The scFv antibody scFv1C9, which was described by us in a previous study, is an effective inhibitor of FGF-1. scFv1C9 blocks the translocation of FGF-1 into the nucleus, resulting in a phenotype similar to the expression of FGF-1 that lacks a nuclear localization signal 20,21. Therefore, scFv1C9 could be an alternative therapeutic for inhibiting tumour growth, potentially through the application of gene delivery methods, lentiviral vectors and electroporation 22–25.

In the present study, we explore potential applications of scFv1C9 in a mouse model through approaches utilizing a lentiviral vector and electroporation.

## Materials and methods

### Cell lines and reagents

The human breast cancer cell lines MDA-MB-231, BT549, MCF-7 and BT474 were obtained from the American Type Culture Collection (ATCC, Manassas, VA, USA) and grown in DMEM (Sigma-Aldrich, St. Louis, MO, USA) supplemented with 10% (v/v) foetal bovine serum (Sigma-Aldrich). The 293FT cell line (Invitrogen, Carlsbad, CA, USA) was maintained in DMEM medium supplemented with 10% (v/v) foetal bovine serum, 0.1 mM MEM non-essential amino acids (Invitrogen), 6 mM L-glutamine (Invitrogen), 1 mM MEM sodium pyruvate (Invitrogen) and 500 μg/ml Geneticin (Invitrogen). SU9516 ([Z]-1,3-dihydro-3-[1H-imidazol-4-ylmethylene]-5-methoxy-2H-indol-2-one), a potent and selective CDK2 inhibitor, was purchased from Tocris Bioscience (Bristol, UK).

### Animals

Female BALB/c nude mice and SCID nude mice of 4–5 weeks of age were obtained from Vital River Company (Beijing, China). Vital River Company also provided Co60-sterilized mouse feed. Water-soluble vitamin C was added to the drinking water to promote the healthy growth of the mice. All experimental procedures were performed in accordance with the guidelines for laboratory animals established by the Northeast Normal University Animal Care and Use Committee.

### Cell treatments

The cells were grown in six-well plates and synchronized in the G0 stage of the cell cycle in DMEM supplemented with 0.1% serum. After 24 hrs of starvation, the cells were stimulated to re-enter active proliferation by incubation with growth medium supplemented with 10% serum. At that time, the cells were treated with SU9516 for 20 hrs. For transfection, the cells were plated on six-well plates at 60% confluency in DMEM supplemented with 10% serum and then transfected with scFv1C9 or the corresponding control plasmid.

### Cell cycle analysis

The cells were fixed and permeabilized in 70% ethanol on ice for 10 min. After washing, the cells were treated with 10 μg/ml RNase A at 37°C for 30 min. Washed cells were incubated with 50 μg/ml propidium iodide (PI) at room temperature for 10 min. The cells were subjected to cell cycle analysis with a flow cytometer (Epics XL-MCL ADC; Beckman: Brea, CA, USA).

### Immunoblotting

The cells were lysed in RIPA buffer (50 mM Tris-HCl pH 7.4, 150 mM NaCl, 1% Triton X-100, 1% sodium deoxycholate, 0.1% SDS, 1 mM EDTA, and 1 μg/ml each aprotinin, leupeptin and pepstatin). Cell lysates were loaded at an equal protein concentration of 30–50 μg/lane and subjected to SDS-PAGE in 10–12% polyacrylamide gels. The samples were then transferred to a PVDF membrane, which was blocked with 5% non-fat milk and incubated overnight at 4**°**C with primary antibodies specific for the following proteins: phospho-p53 (phospho S392; #9281; Cell Signaling Technology: Danvers, MA, USA), p53 (P8999; Sigma-Aldrich: St. Louis, MO, USA), p21 (05-345; Millipore: Billerica, MA, USA), Cyclin E (sc-198; Santa Cruz, Dallas, Texas, USA), CDK2 (sc-6248; Santa Cruz) and actin (A5316; Sigma-Aldrich). Then, the blots were incubated with HRP-conjugated secondary antibodies for 1 hr at room temperature, followed by detection using the ECL reagent.

### Lentivirus for the overexpression of scFv1C9

The three-plasmid-based lentiviral expression system was used in our experiments. The lentiviral shuttle plasmid pLV-UbC-GFP-3FLAG was obtained from Sunbio (Shanghai, China). The pLV-UbC-GFP-3FLAG-*scFv1C9* was produced by inserting *scFv1C9* cDNA into the *EcoR* I site using the in-fusion PCR cloning system. The recombinant vector contained an expression cassette for an scFv1C9 fusion protein. The viruses were propagated in 293FT cells by co-transfecting pLV-UbC-GFP-3FLAG or pLV-UbC-GFP-3FLAG-*scFv1C9* with the psPAX2 and pMD2.G plasmids according to a standard method 26. A real-time PCR assay was used to determine the titre of the recombinant virus by a thermal cycler (ABI 7500, Applied Biosystems, ABI: Carlsbad, CA, USA) The following WPRE-specific primers were used: forward 5′-CCTTTCCGGGACTTTCGCTTT-3′ and reverse 5′-GCAGAATCCAGGTGGCAACA-3′.

### RNAi lentivirus system

Two RNAi target sequences for FGF-1 and VEGF were designed with the Invitrogen RNAi Designer. The target sequences were the following: FGF-1 (588) GCCAGAAAGCAATCTTGTT, FGF-1 (602) GGACTCACTATGGCCAGAA, VEGF (1184) GCAGCTACTGCCATCCAAT and VEGF (1359) GCGGATCAAACCTCACCAA.

The shRNA sequences were determined according to the target sequence and cloned into the pLL3.7 plasmid at *Hpa* I and *Xho* I sites. To confirm the gene-silencing efficiency of FGF-1 and VEGF, each gene-containing plasmid was separately transfected in MCF-7 cells. The cell lysates were used for the analysis of FGF-1 or VEGF expression by western blot. The lentiviruses were packaged in 293FT cells by co-transfecting the shRNA plasmid with the psPAX2 or pMD2.G plasmid. After titration, the virus was used to infect the desired cells.

### Transduction of human breast cancer cells

Approximately 5 × 10^5^ MCF-7 cells were seeded in a 6-well plate, and 2 ml of viral solution (MOI = 10) with 10 μg/ml polybrene was added for 12 hrs. After the infection, the solution was replaced with fresh complete medium. Three days later, the efficiency of transduction was assessed by the GFP expression level. Two cell lines (MCF-7/GFP and MCF-7/GFP-scFv1C9) were established through lentiviral infection. Using the same method above, we produced MDA-MB-231 cells expressing scrambled shRNA, shFGF-1, shVEGF or scFv1C9.

### Tumour formation in nude mice

Experiments were performed with 6-week-old female non-obese diabetic/severe combined immunodeficient (NOD/SCID) mice weighing ∼20 g (*n* = 9). Both MCF-7/GFP and MCF-7/GFP-scFv1C9 cells were grown in 10-cm dishes in DMEM and gently harvested by washing with PBS. The cells were centrifuged and re-suspended into an appropriate volume of medium to produce 5 × 10^6^ cells/0.1 ml. An equal volume of the cell suspension was inoculated subcutaneously in the upper-left quadrant of the mice. The formation of a solid tumour was observed, and the largest (L) and smallest (S) diameters of the tumour were measured once a week. The ability to induce a breast tumour was assessed by determining the tumour incidence and volume.

### Electroporation and luciferase activity analysis

The luciferase-encoding plasmid (pGL3), encoding the luciferase protein under the control of an SV40 promoter, was purchased from Promega (Madison, WI, USA). Approximately 5 × 10^6^ MCF-7 cells were inoculated subcutaneously into the upper-left quadrant of female NOD/SCID mice. The mice were randomly divided into four groups when the tumours grew to ∼5 mm in diameter. The mice were anaesthetized with pentobarbital sodium, and the tumours were injected with the pGL3 plasmid by electroporation (50 μg in 50 μl of PBS). Square-wave electric pulses were generated with an Electro Square Porator T830 (BTX Harvard Apparatus; Holliston, MA, USA). The electroporation pulses were delivered with Tweezertrodes with a distance of ∼5 mm between electrodes, followed by eight square-wave electric pulses 10,24. The application was performed with a set of four pulses in one polar direction and a second set with the opposite polarity. Forty-eight hours after electroporation, the tumour was isolated and dissolved in 200 μl of Luciferase Cell Lysis Buffer (Promega, San Luis Obispo, CA, USA). The supernatants were collected for luciferase activity analysis. Each diluted sample of 25 μl was added to 100 μl of substrate and assayed for luciferase activity in a luminometer (TD-20/20; Turner BioSystems: Sunnyvale, CA, USA). The total protein content for each sample was determined according to the Bradford method using BSA as a standard; absorption readings were measured at 595 nm using a microplate reader. The results were normalized to yield luciferase activity in LUC/mg protein.

### Tumour growth studies

The pEGFP-C1 plasmid was obtained from Clontech Corp (Palo Alto, CA, USA). The pEGFP-*scFv1C9* plasmid, which encodes the EGFP-scFv1C9 protein, was constructed as described in our previous study 20. The tumour-bearing NOD/SCID mice were randomly divided into three groups (*n* = 3) when the tumours grew to ∼5 mm in diameter. The mice were anaesthetized with pentobarbital sodium, and the tumours were injected with PBS (50 μl), pEGFP-C1 plasmid (50 μg in 50 μl of PBS) or pEGFP-*scFv1C9* plasmid (50 μg in 50 μl of PBS). Eight pulses were delivered at 100 V/50 msec. These electroporation parameters were selected based on the results of the luciferase activity experiments. The electroporation was repeated four times at 3-day intervals. The largest (L) and smallest (S) diameters of the tumours were measured once a week.

### *In vivo* lung metastasis model

The MDA-MB-231 Scramble, shFGF-1, shVEGF or scFv1C9 cancer cells were injected into the lateral tail vein of BALB/C nude mice. At 4 weeks post-transplantation, the mice were subjected to preliminary perfusion and killed. The lungs were isolated and fixed in 4% paraformaldehyde for 48 hrs at 4°C. The superior lobe of the right lung was embedded in paraffin and cut into 2-μm sections; the other lobes were subjected to dehydration with 35% sucrose for 1 week at room temperature. The dehydrated tissues were embedded in O.C.T. compound and sectioned with a cryotome.

### Immunohistochemistry

For Ki-67 and CD31 immunohistochemistry, paraffin-embedded 2-μm-thick tissue sections were stained using the protocol supplied by the S-P immunohistochemistry kit (Maixin, Fuzhou, China). To analyse the lung metastasis, the 2-μm paraffin and 20-μm frozen sections were stained with anti-GFP antibody following the Maixin's protocol. The sections were photographed with a Nikon microscope (Eclips TE2000-U; Nikon: Tokyo, Japan).

### Statistical analysis

The Ki-67-positive cells were counted in nine randomly selected fields. The Ki-67 index is the percentage of Ki-67-positive cells as a fraction of the total cells. The CD-31-positive structures were measured with Image J. The microvessel density (MVD) is the percentage of CD-31-positive structures per total area. The results were expressed as the mean ± SD. For the statistics of micrometastases, four slices from one mouse were stained and evaluated for lung micrometastases to assess the ability of the cells to form a lung metastasis. Statistical analysis was performed with the unpaired Student's *t*-test. *P* < 0.05 was considered to be significant. *P* < 0.01 was considered to be highly significant.

## Results

### Expression of scFv1C9 in MCF-7 human breast cancer cells by lentivirus infection

**Figure 1:**
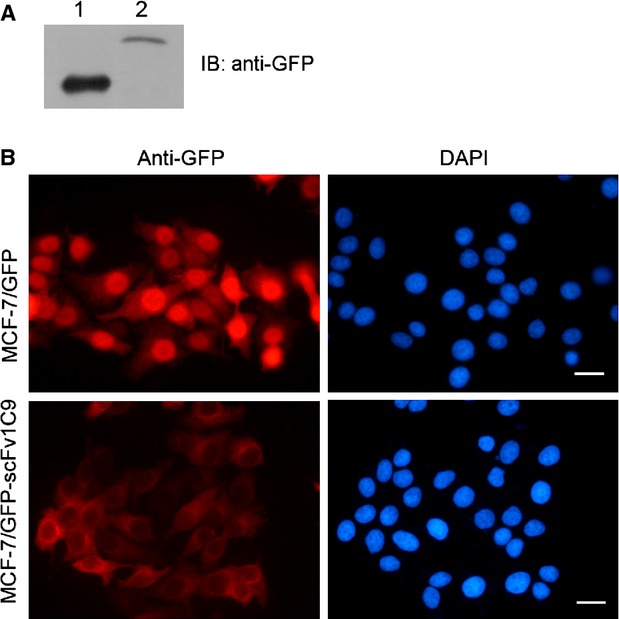
Lentiviral expression of scFv1C9 in MCF-7 cells. GFP-expressing and GFP-scFv1C9-expressing viruses produced in 293FT cells were used to infect MCF-7 cells. At 72 hrs after infection, the cells were screened in medium containing an appropriate concentration of puromycin. (**A**) Western blot analysis shows the expression of GFP-scFv1C9 in MCF-7 cells. Lanes 1 and 2 are the lysates from the MCF-7/GFP and MCF-7/GFP-scFv1C9 cells, respectively. (**B**) Immunostaining with anti-GFP antibody showed that the expression of GFP and GFP-scFv1C9 in MCF-7 cells (scale bar, 10 μm).

In our previous study, we reported that scFv1C9 effectively inhibits FGF-1 by blocking the intracrine FGF-1 pathway, thereby decreasing the proliferation of breast carcinoma cells 20. In this study, the MCF-7/GFP and MCF-7/GFP-scFv1C9 cell lines were established by lentivirus infection. Western blot and immunostaining with anti-GFP antibody confirmed the expression of GFP-scFv1C9 in MCF-7 cells (Fig.1A and B).

### Targeting FGF-1 with scFv1C9 reduced tumourigenesis

**Figure 2:**
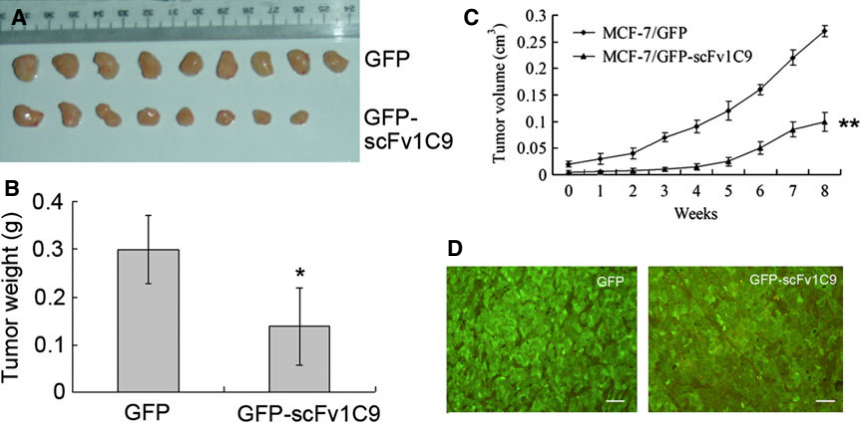
Targeting FGF-1 with scFv1C9 reduced tumourigenesis. Mice were inoculated subcutaneously with the MCF-7/GFP or the MCF-7/GFP-scFv1C9 cells in the upper-left quadrant. Four weeks after inoculation, the tumour size was first measured. The measurements were repeated once a week until the mice were killed during the 12th week. (**A**) The tumours isolated from the mice 12 weeks after inoculation. (**B**) The statistical analysis of the tumour weight. (**C**) The tumour volume of the MCF-7/GFP or MCF-7/GFP-scFv1C9 tumours in SCID mice (the first data point represents the initial measurement 4 weeks after the inoculation). (**D**) The expression of GFP or GFP-scFv1C9 was verified by immunohistochemical staining of the tumour tissues with anti-GFP antibody (scale bar, 50 μm).

Our earlier work showed that the expression of scFv1C9 prevented FGF-1 from entering the nucleus and caused G1 arrest through p21 up-regulation 20. Next, we asked whether the retention of FGF-1 in the cytoplasm could reduce tumourigenesis. Female NOD/SCID mice were subcutaneously inoculated with equal numbers of MCF-7/GFP or MCF-7/GFP-scFv1C9 cells (5 × 10^6^) in the upper-left quadrant. Four weeks after inoculation, all nine mice in the MCF-7/GFP group had formed tumours, whereas only two of the nine mice that had been inoculated with MCF-7/GFP-scFv1C9 had a tumour (Table1). The tumour size was measured once a week until the mice were killed and the tumours were isolated in the 12th week. The weight of GFP-scFv1C9-overexpressing tumours was significantly lower than that of tumours from the GFP group (Fig.2A and B). Tumour volume was calculated according to the formula: V = S^2^*L/2. The results indicated that the growth of the MCF-7/GFP-scFv1C9 tumours was much slower than that of the MCF-7/GFP group (Fig.2C). The expression of scFv1C9 in the tumour tissue was confirmed by immunohistochemical staining (Fig.2D).

**Table 1:** Tumourigenicity of MCF-7 cells transfected with GFP or GFP-scFv1C9

Groups	x	x	Weeks
X	4	8	12
MCF-7/GFP	9/9	9/9	9/9
MCF-7/GFP-*scFv1C9*	2/9	5/9	8/9

### scFv1C9 up-regulated p21 expression and arrested the cell cycle

**Figure 3:**
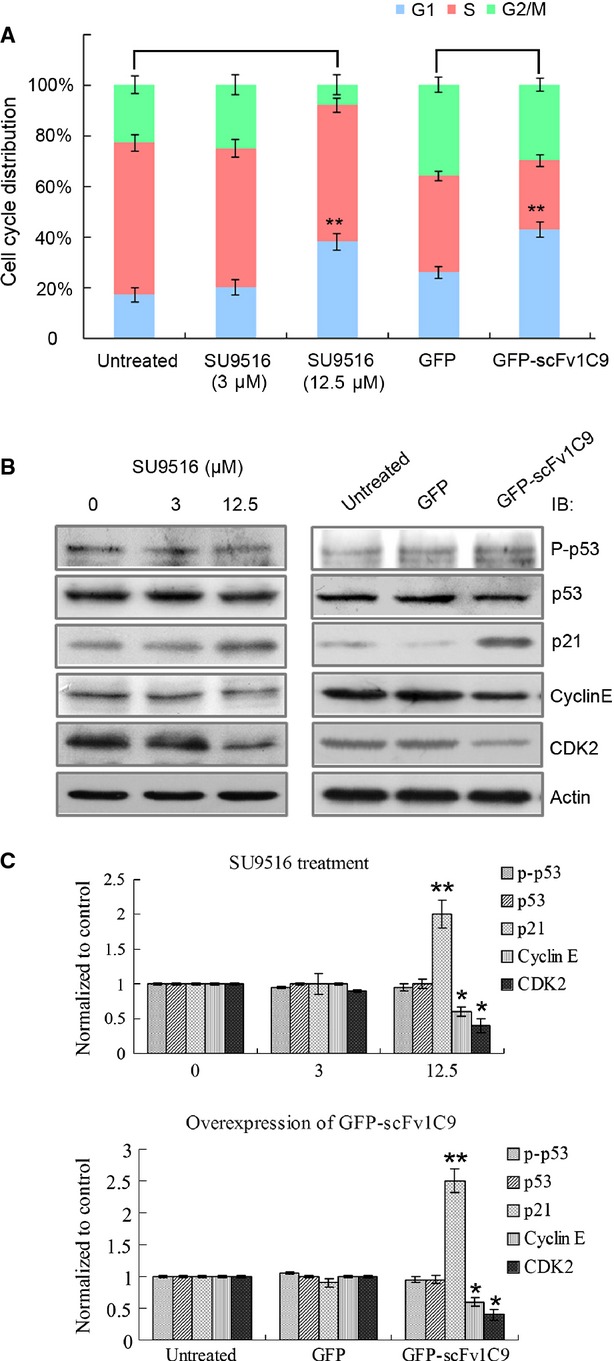
scFv1C9 up-regulated p21 expression and arrested the cell cycle. (**A**) The addition of 12.5 μM SU9516 significantly increased the cell number of MCF-7 cells in the G0/G1 stage compared with untreated cells or cells incubated with 3 μM SU9516. A similar extent of G0/G1 cell cycle arrest was observed in scFv1C9-overexpressing cells. (**B**) After incubation with SU9516 or scFv1C9, MCF-7 cell lysates were immunoblotted for cell cycle-related proteins. MCF-7 cells overexpressing scFv1C9 exhibited a clear increase in the expression of p21 and a slight decrease in Cyclin E and CDK2 expression, behaviour that is consistent with SU9516 treatment. (**C**) The statistical analysis of the relative protein level.

To explore the underlying mechanisms responsible for the scFv1C9-induced inhibition of tumour growth, a cyclin-dependent kinase 2 (CDK2) inhibitor, SU9516, was used as a positive control in the treatment of cells. The overexpression of scFv1C9 resulted in an arrest in the G0/G1 phase, similar to the effect of SU9516 (Fig.3A). Further evidence revealed that the overexpression of scFv1C9 elicited an obvious increase in the expression of p21 and decrease in Cyclin E and CDK2, both of which are consistent with SU9516 incubation (Fig.3B and C).

### Targeting FGF-1 with scFv1C9 reduced the lung metastasis of breast cancer

**Figure 4:**
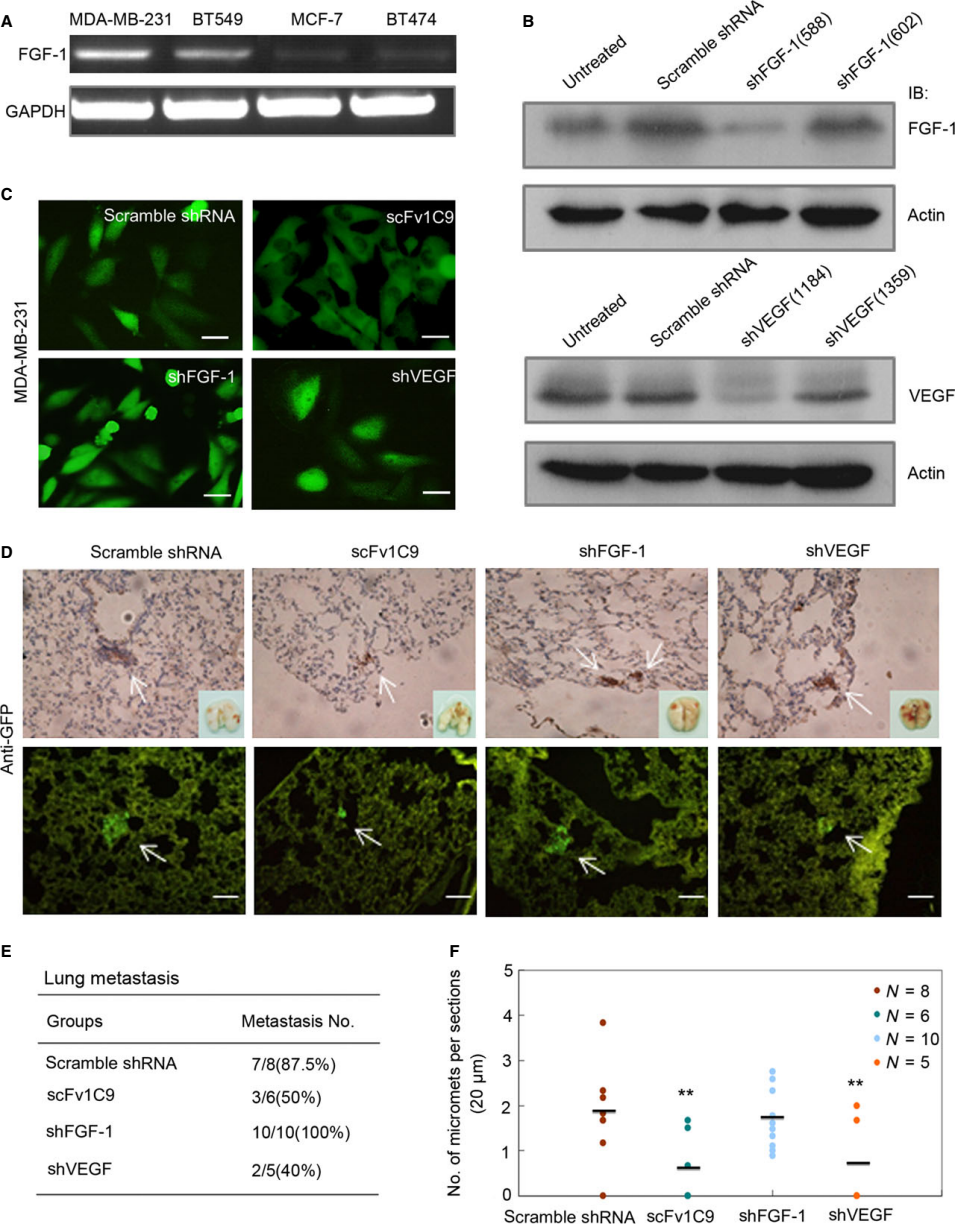
scFv1C9 inhibited the metastasis of breast cancer to the lung. (**A**) RT-PCR of FGF-1 in the MDA-MB-231, BT549, MCF-7 and BT474 malignant breast cancer cell lines. FGF-1 is highly expressed in highly metastatic breast cancer cells such as MDA-MB-231 cells. (**B**) Western blot analysis of the silencing efficiency of shFGF-1 and shVEGF. (**C**) MDA-MB-231 cells expressing Scramble shRNA, shFGF-1, shVEGF or scFv1C9 (scale bar, 10 μm). (**D**) Anti-GFP antibody-stained sections of lungs isolated from mice were evaluated microscopically at 4 weeks post-intravenous implantation. The micrometastases were diffusely distributed in the centre or periphery of lung tissue in the paraffin sections and cryosections. The arrows indicate clusters of metastatic cells (scale bar, 100 μm). (**E**) Number of lung micrometastases per section in individual mice. scFv1C9 and shVEGF inhibited metastasis of breast cancer to the lung of MDA-MB-231 cells. (**F**) Incidence of lung metastasis in mice that had been injected with cells expressing scramble shRNA, scFv1C9, shFGF-1 or shVEGF.

Next, we sought to determine whether scFv1C9 affects breast cancer metastasis. First, we assessed the FGF-1 mRNA levels in various breast cancer cells (MDA-MB-231, BT549, MCF-7, and BT474). FGF-1 mRNA was much more abundant in the MDA-MB-231 cells compared to the other cell types, which was consistent with the strong ability of MDA-MB-231 cells to form lung metastases (Fig.4A). The MDA-MB-231 Scramble, shFGF-1, shVEGF or scFv1C9 cancer cells were established by lentivirus infection of MDA-MB-231 cells (Fig.4B and C). The cells were injected intravenously into mice to observe the formation of lung metastases. Four weeks after inoculation, the lung tissues were isolated to analyse the metastases. Seven of the eight mice in the scramble group had formed lung micrometastases, whereas only three of the six mice that had been inoculated with MDA-MB-231/GFP-scFv1C9 and two of the five mice that had been inoculated with MDA-MB-231/shVEGF had developed slight lung metastases (Fig.4D and E). However, all mice that had been inoculated with MDA-MB-231/shFGF-1 formed lung micrometastases. Next, the number of metastases was counted in sections of the lung lobe. The overexpression of scFv1C9, similar to shVEGF, resulted in the formation of fewer micrometastases when compared to the controls (Fig.4F).

### Electroporation-gene therapy with *scFv1C9* delayed tumour growth

**Figure 5:**
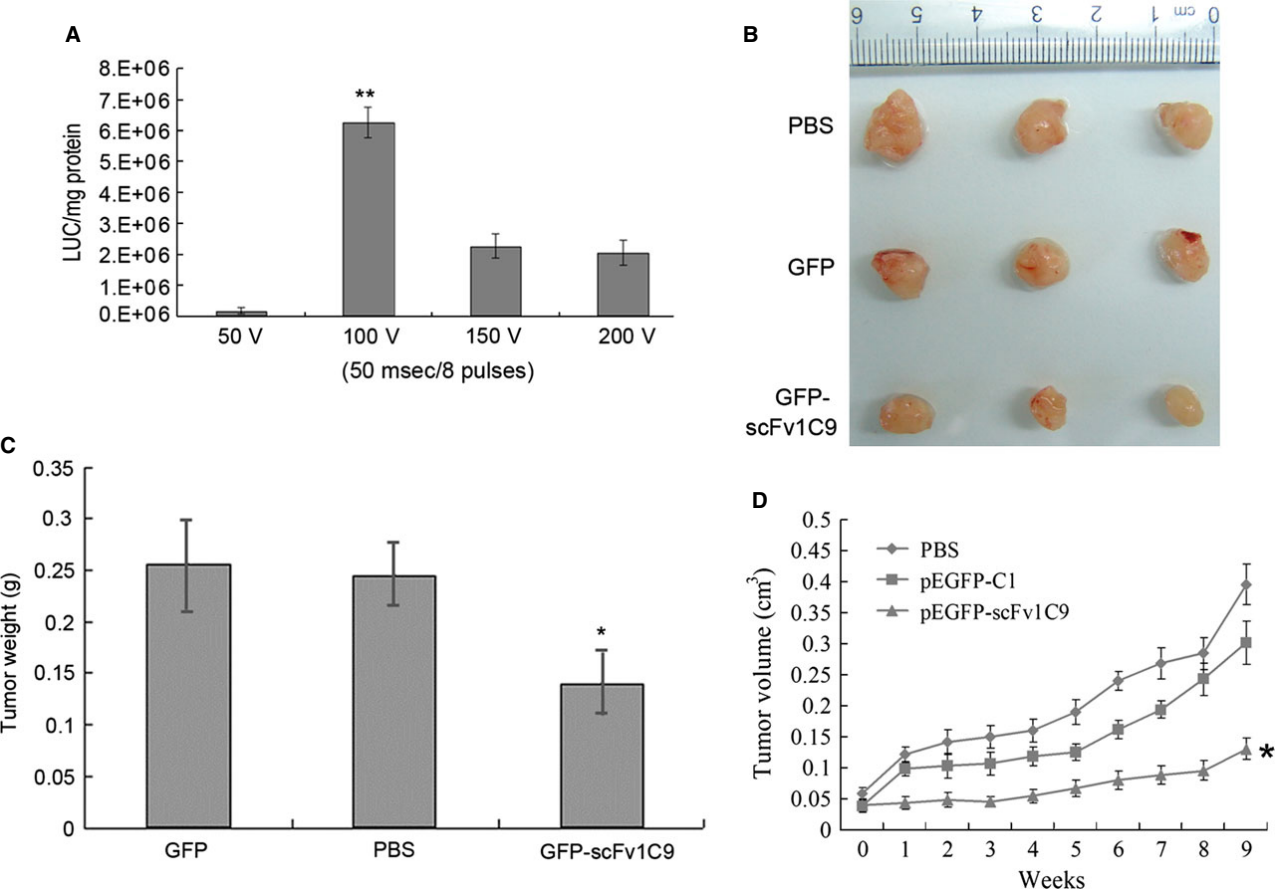
Electroporation gene therapy with *scFv1C9* delayed tumour growth. The SCID mice bearing MCF-7 tumours were electroporated with PBS, pEGFP-C1 plasmid or pEGFP-*scFv1C9* plasmid under optimized conditions. The electroporation was repeated four times at 3-day intervals. The tumour size was determined once a week using callipers. Nine weeks after the first treatment, the mice were killed, the tumours were isolated and paraffin-embedded sections were prepared. (**A**) The expression level of luciferase in the MCF-7 tumours. (**B**) Tumours isolated from mice after treatment. (**C**) Statistical analysis of the tumour weight. (**D**) The tumour growth curve of tumours electroporated with PBS, pEGFP-C1 or pEGFP-*scFv1C9*.

To explore the therapeutic applications of scFv1C9 *in vivo*, the gene transfer technique of electroporation was used to deliver the *scFv1C9* gene. The appropriate plasmid was injected into the tumour in the mice, and electric pulses were subsequently applied to promote DNA transfer. To optimize the electroporation voltage, the efficacy of the electrotransfer was assessed first with a luciferase-encoding plasmid that was injected into a solid tumour. Electric pulses were then immediately delivered through tweezer electrodes. To optimize the experimental conditions, electroporation pulses were set as at 50 V/50 msec., 100 V/50 msec., 150 V/50 msec. and 200 V/50 msec. The luciferase activity was analysed with the lysates from tumour tissues after 2 days. The results showed that the luciferase activity was 1.65 × 10^5^ ± 0.1 × 10^5^, 6.26 × 10^6^ ± 0.5 × 10^6^, 2.26 × 10^6^ ± 0.4 × 10^6^ and 2.04 × 10^6^ ± 0.4 × 10^6^ LUC/mg protein at 50 V/50 msec., 100 V/50 msec., 150 V/50 msec., and 200 V/50 msec., respectively (Fig.5A). Higher expression of luciferase was observed with 100 V/50 msec. or eight pulses, which was chosen as the optimal set of conditions for the subsequent experiments.

The final goal of this research was to explore whether scFv1C9 has substantial benefits for tumour therapy. Therefore, SCID mice bearing MCF-7 tumours (4 weeks after inoculation, *n* = 3) were injected intratumourally with PBS alone (50 μl), the pEGFP-C1 plasmid (50 μg in 50 μl of PBS) or the pEGFP-*scFv1C9* plasmid (50 μg in 50 μl of PBS). Subsequently, electroporation was applied as eight pulses delivered at 100 V/50 msec., five times at 4-day intervals. The tumour size was determined once a week using callipers. In the 13th week after inoculation, the mice were killed, and the tumours were isolated. The weight of the pEGFP-*scFv1C9* electroporated tumours was significantly lower compared than that of the pEGFP-C1 electroporated tumours and the PBS electroporated tumours (Fig.5B and C). The relative tumour growth rates in the different groups over the 9 weeks from the first treatment are shown in Figure5D. Electroporation with the control plasmid (pEGFP-C1) did not result in a significant growth delay compared with the PBS control, whereas electroporation with the *pEGFP-scFv1C9* plasmid resulted in a significant growth delay compared with both the control plasmid (pEGFP-C1) and blank (PBS) groups.

### Ki-67 index and MVD were significantly decreased

**Figure 6:**
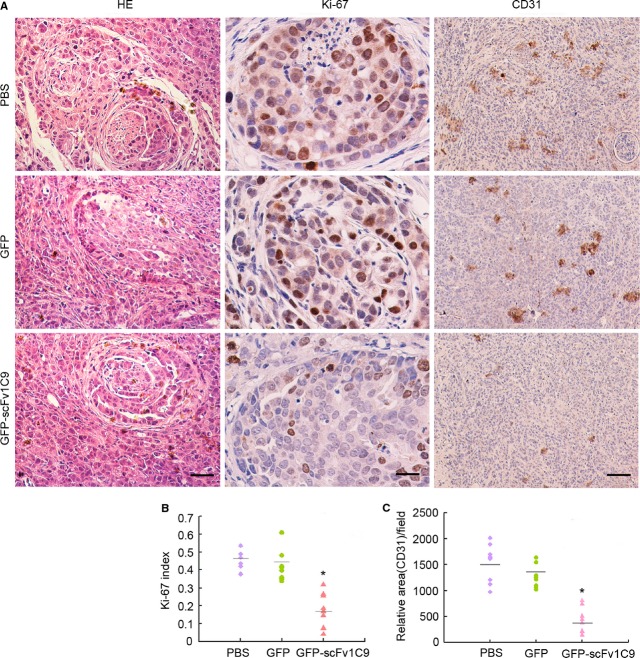
The Ki-67 index and microvessel density were significantly decreased upon the expression of scFv1C9. The tumour sections were stained with haematoxylin-eosin (HE) and antibodies specific for Ki-67 and CD31. Representative images of stained sections are shown. HE (scale bar, 50 μm), Ki-67 (scale bar, 10 μm), and CD31 (scale bar, 50 μm). (**A**) Representative images. (**B**) Statistical analysis of the Ki-67 index. (**C**) Statistical analysis of the microvessel density.

To investigate the tumour growth delay induced by the electroporation of pEGFP-*scFv1C9*, the tumour sections were analysed for immunohistochemical staining for Ki-67 (frequently used as a marker of proliferative activity) and CD31 (a marker for vascular endothelial cells). The Ki-67 index of the PBS and the pEGFP-C1 mouse groups was 47.6 ± 5.6% and 41.3 ± 8.5%, respectively, but was only 17.3 ± 9.6% for the pEGFP-*scFv1C9* group, which was significantly lower than in the PBS and pEGFP-C1 groups (*P* < 0.05). The relative MVD of the PBS and the pEGFP-C1 groups was 15,301 ± 3543 and 12,565 ± 2083 per μm^2^, respectively, but was only 4206 ± 2420 per μm^2^ in the pEGFP-*scFv1C9* group (Fig.6), significantly lower than in the control groups.

## Discussion

Under physiological conditions, FGF-1 plays important roles in cell proliferation and angiogenesis 27,28. However, FGF-1 is overexpressed in many types of cancer tissue, such as bladder cancer, hepatocellular carcinoma, pancreatic cancer and breast cancer 3,11,25. MCF-7 cells that stably express FGF-1 facilitate the growth and metastasis of tumours when transplanted into nude mice 29,30, indicating that FGF-1 plays important roles in the pathological processes of cancer. The single-chain antibody scFv1C9 is an effective inhibitor of FGF-1: this antibody induces cell cycle G0/G1 arrest by up-regulating p21, a potential inhibitor of the G1 cyclin-dependent kinases that are necessary for the control of the G1 to S phase transition in mammalian mitosis. MCF-7/GFP-scFv1C9 cells have a significantly lower tumourigenicity than MCF-7/GFP cells, suggesting that FGF-1 is important for the development of breast cancer and that blocking FGF-1 with a specific antibody is an effective strategy to inhibit breast cancer. This antibody may be an alternative to PD 173074, a synthetic compound of the pyrido-[2.3-*d*]-pyrimidine class that selectively inhibits FGFR tyrosine kinase activity and autophosphorylation 14,15. PD 173074 anticancer agent has been shown to induce the G0/G1 growth arrest of breast cancer cells, similar to scFv1C9. Both compounds hold promise for the prevention and control of breast cancer.

In contrast to most other growth factors, the FGF-1 contains a NLS, which suggests that FGF-1 may be involved in signalling through an intracrine mechanism in addition to the classic FGF receptor signalling pathway. The regulation of the intracrine pathway is unclear. The function of FGF-1 is usually mediated by FGFRs which have kinase activity *via* the FGFR signalling pathway. In the intracrine pathway, FGF-1 has been reported to interact with p53 and inhibit p53-dependent apoptosis and cell growth arrest 6,23. In our study, we show that scFv1C9 prevents the nuclear translocation of FGF-1 and up-regulates the expression of p21 but has no effect on p53 and the phosphorylation of p53. These results suggest that p53 is not involved in the p21 up-regulation induced by the expression of scFv1C9. There is likely a novel mechanism that regulates FGF-1 nuclear translocation and p21 expression. Further studies will be needed to determine the exact mechanism by which scFv1C9 induces p21 in a p53-independent manner.

FGF-1 has been reported to promote the invasion and metastasis of breast cancer by inducing MMP-9 expression 31. To investigate the role of scFv1C9 in FGF-1-mediated invasion and metastasis, lentivirus-infected cells were injected into the lateral tail vein of mice, and tumour foci were observed during the following 4 weeks. We found that the overexpression of scFv1C9 and the silencing of VEGF reduced the formation of metastases, indicating the important roles of scFv1C9 in inhibiting breast cancer metastasis. However, silencing FGF-1 had no effect on the metastasis of breast cancer. We speculate that this lack of effect is because of the incomplete silencing of FGF-1 or that other sources of FGF-1 are available to stimulate cancerous cell proliferation and metastasis; however, the expression of scFv1C9 was sufficient to block FGF-1 in these cells. These results confirmed that the overexpression of scFv1C9 can inhibit the metastasis of breast cancer to the lung, again suggesting the importance of FGF-1 in the metastasis of breast cancer.

Recently, electroporation has been employed in pre-clinical studies in animal models and in clinical settings for the delivery of chemotherapeutic drugs with very high anti-tumour efficacy and negligible side effects 32. In addition, some recent studies have tested the use of electroporation to deliver plasmid DNA to various types of tissues *in vivo*, such as skin, liver, testis, brain, skeletal muscle and tumours 33. In this study, we delivered scFv1C9 by electroporation of the solid tumour that we had transplanted into mice; significant inhibition of tumour growth and angiogenesis was achieved. The intra-tumoural expression of scFv1C9 was indicated to significantly inhibit the growth of tumour cells and the formation of microvessels in the mouse model. Although further improvements of scFv1C9 by molecular modifications are needed before testing human applications, the scFv1C9 antibody offers promise for inhibiting breast cancer.

In conclusion, the present study demonstrated that scFv1C9 is an effective inhibitor of FGF-1 *in vitro* and *in vivo*. Therefore, scFv1C9 is a potential candidate for inhibiting breast cancer.
